# Multiple B-cell epitope vaccine induces a *Staphylococcus* enterotoxin B-specific IgG1 protective response against MRSA infection

**DOI:** 10.1038/srep12371

**Published:** 2015-07-23

**Authors:** Zhuo Zhao, He-Qiang Sun, Shan-Shan Wei, Bin Li, Qiang Feng, Jiang Zhu, Hao Zeng, Quan-Ming Zou, Chao Wu

**Affiliations:** 1 National Engineering Research Center of Immunological Products, Department of Microbiology and Biochemical Pharmacy, College of Pharmacy, Third Military Medical University, Chongqing 400038, PR China; 2 Department of Gastroenterology, Xinqiao Hospital, Third Military Medical University, Chongqing 400038, PR China; 3 Department of Pathology, Southwest Hospital, Third Military Medical University, Chongqing 400038, PR China

## Abstract

No vaccine against methicillin-resistant *Staphylococcus aureus* (MRSA) has been currently approved for use in humans. *Staphylococcus* enterotoxin B (SEB) is one of the most potent MRSA exotoxins. In the present study, we evaluated the efficacy and immunologic mechanisms of an SEB multiple B-cell epitope vaccine against MRSA infection. Synthetic overlapping peptide ELISA identified three novel B-cell immunodominant SEB epitopes (in addition to those previously known): SEB_31–48_, SEB_133–150_, and SEB_193–210_. Six B-cell immunodominant epitopes (amino acid residues 31–48, 97–114, 133–150, 193–210, 205–222, and 247–261) were sufficient to induce robust IgG1/IgG2b-specific protective responses against MRSA infection. Therefore, we constructed a recombinant MRSA SEB-specific multiple B-cell epitope vaccine Polypeptides by combining the six SEB immunodominant epitopes and demonstrated its ability to induce a robust SEB-specific IgG1 response to MRSA, as well as a Th2-directing isotype response. Moreover, Polypeptides-induced antisera stimulated synergetic opsonophagocytosis killing of MRSA. Most importantly, Polypeptides was more effective at clearing the bacteria in MRSA-infected mice than the whole SEB antigen, and was able to successfully protect mice from infection by various clinical MRSA isolates. Altogether, these results support further evaluation of the SEB multiple B-cell epitope-vaccine to address MRSA infection in humans.

*Staphylococcus aureus* is a facultative human pathogen that causes skin and wound-related infections, food poisoning, septicaemia and pneumonia. The worldwide emergence of methicillin-resistant *S. aureus* (MRSA) infections is responsible for increasing health costs, as well as patient morbidity and mortality^1^,^2^, especially in the United States^3^, China^4^ and Japan^5^, and Germany^6^. Vaccination is a proven, safe and cost-effective way to protect against infectious diseases; however, no licensed prophylactic or therapeutic vaccines against MRSA are currently available^7^,^8^.

MRSA express a large and diverse repertoire of virulence factors, including many toxins and invasive enzymes. Recent study demonstrated that the more toxic MRSA isolates cause more severe disease symptoms^9^. Staphylococcal enterotoxin B (SEB)—one of the most potent staphylococcal enterotoxins (SEs)^10^—plays a vital role in toxic shock syndrome induced by community-acquired MRSA and is produced by a majority of MRSA isolates in very high concentrations^11^,^12^. According to NCBI-BLAST, the SEB protein sequence is conserved across many prevalent MRSA isolates including N315^13^, ST228^14^, Mu3^15^, Mu50^16^, JH1, and JH9^17^. Moreover, previous studies confirm that SEB monoclonal antibodies can partially protect humans from toxic shock syndrome and other *S. aureus*-associated diseases^18^,^19^ and suggest that SEB might be an ideal MRSA vaccine candidate, as confirmed by a previous study in our lab^20^.

Earlier studies used mutant or attenuated SEB as vaccine against lethal SEB challenge or *S. aureus* infection^21^,^22^; however, we have found that the whole antigen is unable to induce the most robust response against pathogen infection for at least two reasons. First, whole antigen vaccines are not as potent as epitope-specific vaccines^23^, since only a few immunodominant epitopes are sufficient to induce a protective response^24^,^25^. Second, *S. aureus* is highly adept at evading opsonisation. Thus, since previous studies have confirmed that immunodominant SEB peptide vaccination can convey a potent humoral immune response^26^, this methodology may aid in the optimisation of MRSA vaccine strategy. Similar epitope-based approaches in other subunit vaccines currently used for HIV^27^, respiratory syncytial virus^28^, and Helicobacter pylori^29^.

In the present work, we have mapped the B-cell immunodominant epitopes in SEB by synthetic overlapping peptide ELISA, combined these immunogens to construct a single SEB-specific multiple B-cell epitope vaccine (Polypeptides), and confirmed the vaccine’s ability to stimulate the synergetic opsonophagocytosis of MRSA bacteria and protect mice from infection by various clinical MRSA isolates.

## Results

### Identification of the immunodominant linear B-cell epitopes in MRSA SEB

**Figure 1:**
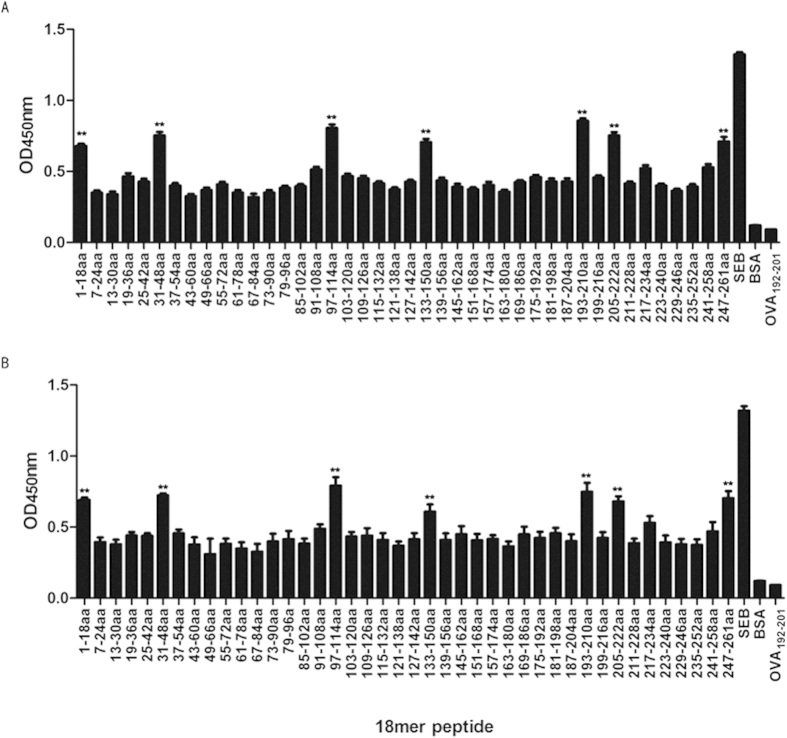
*Staphylococcus* enterotoxin B (SEB) B-cell epitope mapping. B cell epitope mapping of SEB using an overlapping 18-mer peptide ELISA. To determine the immunodominant peptides of SEB, microtiter plates were coated with synthetic overlapping peptides that spanned the entire length of the SEB of MRSA252 or BSA and OVA_192–201_ (negative control peptides). Then, sera samples from BALB/c mice that were directly infected with MRSA252, and that were immunised with rSEB plus AlPO_4_ adjuvant before MRSA252 infection were detected (The antisera were diluted to 1:300). The absorbance was read at 450 nm. The raw O.D. values shown were obtained using serum (diluted by a factor of 1:300) from three independent experiments assayed concurrently. Data are represented as the means ± SEM. Probability values of p < 0.05 were considered significant and are denoted by an asterisk (*). **p < 0.01. (**A**) Antiserum samples from BALB/c mice immunised with rSEB plus AlPO_4_ adjuvant following infection with MRSA252 were detected for the immunodominant response. (**B**) Antiserum samples from non-immunised BALB/c mice infected with MRSA252 were detected for the immunodominant response.

We previously produced recombinant (rSEB) that inhibited the ability of native SEB to induce T-cell mitogenesis and cytokine production in BALB/c splenocytes, and also identified three immunodominant SEB B-cell epitopes SEB_97–114_, SEB_205–222_ and SEB_247–261_ using the rSEB antisera^20^. However, we had suggested that there might be other SEB B-cell epitope-specific response during MRSA infection that were different with rSEB immunization. In the present study, linear B-cell epitope mapping of SEB was further determined by an ELISA with overlapping 18-mer peptides and antisera obtained from rSEB-immunised mice following MRSA challenge. The results indicated that the antisera’s strongest IgG antibody reactivity concentrated on seven major immunodominant peptides: SEB_1–18_, SEB_31–48_, SEB_97–114_, SEB_133–150_, SEB_193–210_, SEB_205–222_ and SEB_247–261_ , and the absorbances at 450 nm for these peptides were significantly higher than BSA (P < 0.01) and higher than OVA_192–201_(P < 0.01) (Fig. 1). Among the seven immunodominant peptides, three peptides—SEB_31–48_, SEB_133–150_ and SEB_193–210_—were novel epitopes that have not been reported previously, and the SEB_1–18_ fragment comprises an N-terminal localisation signal peptide.

### Immunisation with individual immunodominant SEB epitopes elicited a protective response against MRSA challenge

**Figure 2:**
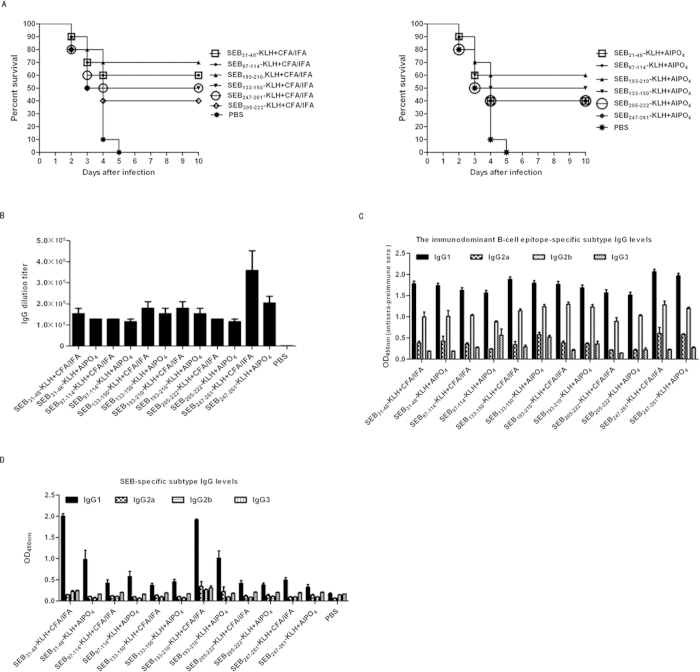
The protective role of the individual immunodominant SEB epitopes against MRSA252 challenge. (**A**) Percent survival in mice immunised with individual epitopes plus AlPO_4_ or CFA/IFA adjuvant, AlPO_4_ or CFA/IFA adjuvant alone, or PBS alone. The significance of protective immunity generated by the individual epitope vaccine was measured with Fisher’s exact test: : for CFA/IFA adjuvanted vaccine, SEB_31–48_-KLH (P = 0.011), SEB_97–114_-KLH (P = 0.011), SEB_133–150_-KLH (P = 0.033), SEB_193–210_-KLH (P = 0.003), SEB_205–222_-KLH (P = 0.087) SEB_247–261_-KLH (P = 0.033); for AlPO_4_ adjuvanted vaccine, SEB_31–48_-KLH (P = 0.033), SEB_97–114_-KLH (P = 0.087), SEB_133–150_-KLH (P = 0.033), SEB_193–210_-KLH (P = 0.011), SEB_205–222_-KLH (P = 0.087), SEB_247–261_-KLH (P = 0.087). (**B**) Antibody production of Polypeptides or the individual immunodominant epitope -vaccinated mice. ELISA detection of the individual immunodominant epitope specific antibody levels in mice immunised by the individual immunodominant epitope. (**C**) ELISA detection of Polypeptides specific antibody subtype in mice immunised with the individual epitopes. IgG subclass distribution in mice immunised with different formulations. Results are representative of three experiments and data are mean ± SD of samples from ten mice. (**D**) ELISA detection of SEB specific antibody subtype in mice immunised with the individual epitopes. IgG subclass distribution in mice immunised with different formulations. Results are representative of three experiments and data are mean ± SD of samples from ten mice.

To determine the protective role of the individual immunodominant epitopes against MRSA infection, BALB/c mice were immunised with the keyhole limpet hemocyanin (KLH)-conjugated epitopes plus Freund (CFA/IFA) and AlPO_4_ adjuvants, AlPO_4_ alone, or PBS alone prior to MRSA252 infection. In total, 70% or 60% of mice immunised with SEB_193–210_-KLH plus CFA/IFA or AlPO_4_ adjuvant survived MRSA challenge without exhibiting any clinical symptoms, respectively. Moreover, the survival rates of SEB_193–210_-KLH plus CFA/IFA (70%) immunised mice were higher than those of mice immunised with SEB_193–210_-KLH plus AlPO_4_ adjuvant (60%), SEB_31–48_-KLH plus CFA/IFA (60%), SEB_31–48_-KLH plus AlPO_4_ (50%), SEB_97–114_-KLH plus CFA/IFA (60%), SEB_97–114_-KLH plus AlPO_4_ (40%), SEB_133–150_-KLH plus CFA/IFA (50%), SEB_133–150_-KLH plus AlPO_4_ (50%), SEB_247–261_-KLH plus CFA/IFA (50%), SEB_247–261_-KLH plus AlPO_4_ (40%), and SEB_205–222_-KLH plus CFA/IFA (40%), SEB_205–222_-KLH plus AlPO_4_(40%) (Fig. 2A). Conversely, all PBS only mock-immunised died within 5 days of challenge. These results showed that all of the individual conjugated epitopes induced partial protective role against MRSA infection, where SEB_193–210_ elicited the strongest protective response when given with CFA/IFA or AlPO_4_ adjuvants. It could be noted that in present study, we only analysed six of the immunodominant B-cell epitopes. The seventh, SEB_1–18_, is the signal peptide that is usually cleaved from the mature peptide and could not be used for MRSA vaccine.

To measure the individual epitope-specific antibody titres, the sera of immunised mice were harvested 1 week after the last booster immunisation and used for ELISA assays. As expected, all vaccinated groups exhibited increased levels of specific IgG. The mean antibody titre in the Polypeptides plus CFA/IFA or AlPO_4_ adjuvant vaccine-immunised group was up to 6.73 × 10^5^- and 4.69 × 10^5^-fold higher, respectively, than that in the PBS group (Fig. 2B).

Serum IgG subclass analysis in the individual epitope-vaccinated mice revealed an IgG1/IgG2b isotype response, which was found to be similar with all formulations (Fig. 2C). The isotype ratio (IgG1/IgG2b) was found to be >1.0, whereas IgG3 levels were negligible in all samples analysed. Thus, the specific increase in the IgG1 isotype (P < 0.01) is indicative that IgG1-biased responses may be more potent in neutralising SEB toxin (Fig. 2D).

### Polypeptides immunisation protected mice against lethal MRSA challenge

**Figure 3:**
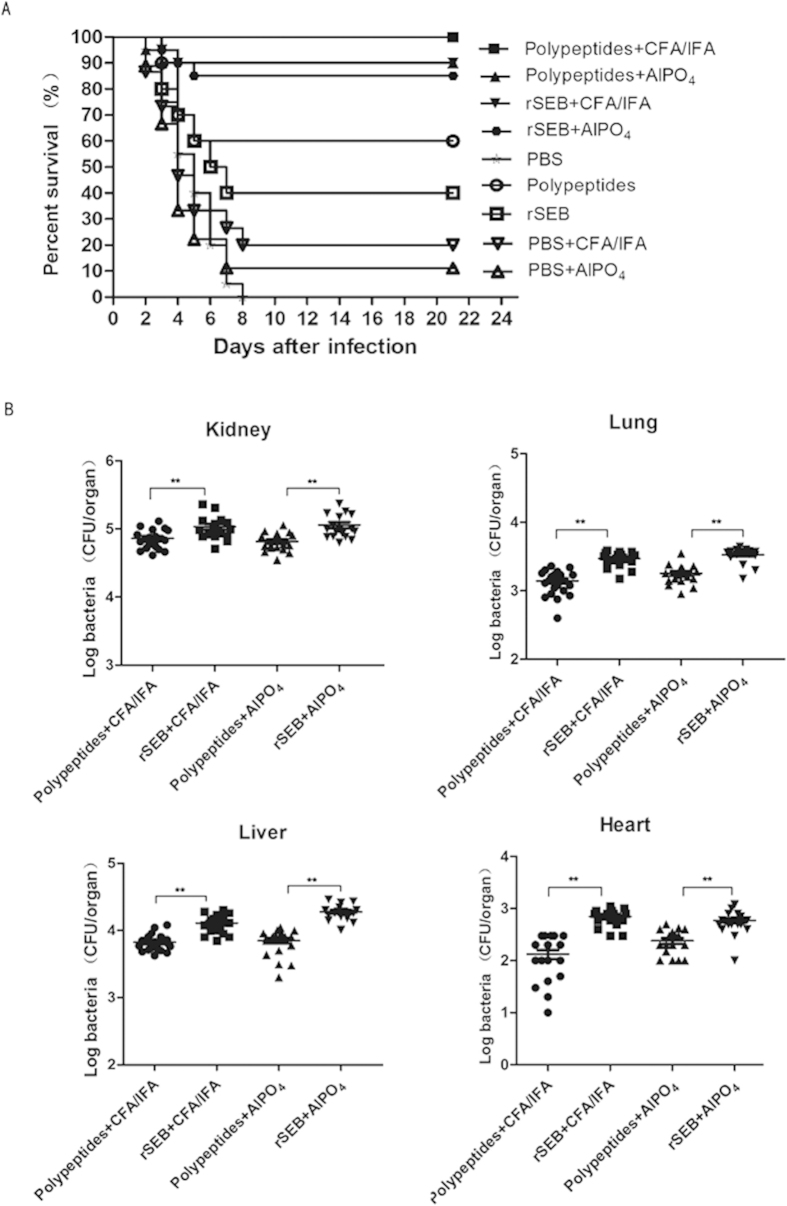
The multiple epitope-vaccine induced protective response against MRSA252 infection in the immunised mice. (**A**) Percent survival against MRSA252 infection in the immunised mice. (**B**) Bacterial burden in the organs of mice after challenge with MRSA 252. **P < 0.01(N = 10; independent experiments).

To determine the protective role of Polypeptides against MRSA infection, BALB/c mice were immunised with Polypeptides or rSEB plus AlPO_4_ or CFA/IFA adjuvant, Polypeptides or rSEB alone, AlPO_4_ or CFA/IFA adjuvant alone, or PBS alone prior to lethal MRSA252 challenge. Notably, 100% or 90% of mice immunised with Polypeptides plus CFA/IFA or AlPO4 adjuvant survived without clinical symptoms, which was higher than that in rSEB plus CFA/IFA or AlPO_4_ adjuvant (90% and 85%), the Polypeptides or rSEB alone groups (60% and 40%), the CFA/IFA or AlPO_4_ adjuvant only groups (30% and 20%), and the PBS group (0% and 0%), respectively (Fig. 3A). While more robust protection was observed in the Polypeptides group as compared to the rSEB group, this finding failed to reach statistical significance.

To further confirm the effect of Polypeptides against MRSA challenge, the organs of immunised mice were assessed for bacterial load after challenge with MRSA252. As expected, Polypeptides-immunised mice displayed a significantly lower bacterial burden than those of rSEB-immunised and PBS-control counterparts (Fig. 3B).

**Figure 4:**
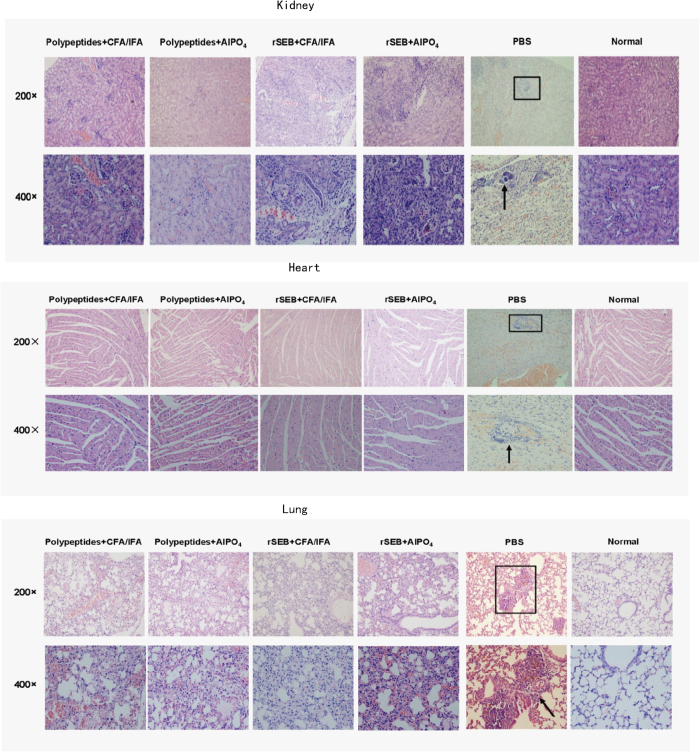
Histological analysis of MRSA challenged, Polypeptides-immunised mice revealed normal physiological architecture. Hematoxylin and eosin staining of kidney sections at day 21 after infection. Microscopic images of kidneys, hearts and lungs, 200 × (top row) and 400 × (bottom row). Abscess formation or scattered colonies of bacteria were only found in mock-immunised animals (black arrow).

Histological analysis of MRSA challenged, Polypeptides-immunised mice revealed normal physiological architecture of the myocardial fibres, renal tubules, and pulmonary alveolar with no detectable bacterial colonies, while rSEB-immunised mice exhibited slight changes in the physiological architecture of pulmonary alveolar (fibrin and serous effusion) and renal tubules (neutrophils). In comparison, scattered bacterial colonies were readily observed in the heart, kidney, lung, and liver of PBS control mice, particularly in the kidney abscesses (Fig. 4). Taken together, these experiments indicated that Polypeptides immunisation provides a more robust protection against MRSA challenge than conveyed by the entire rSEB antigen.

### Polypeptides immunisation induced an SEB-specific IgG1 protective response

**Figure 5:**
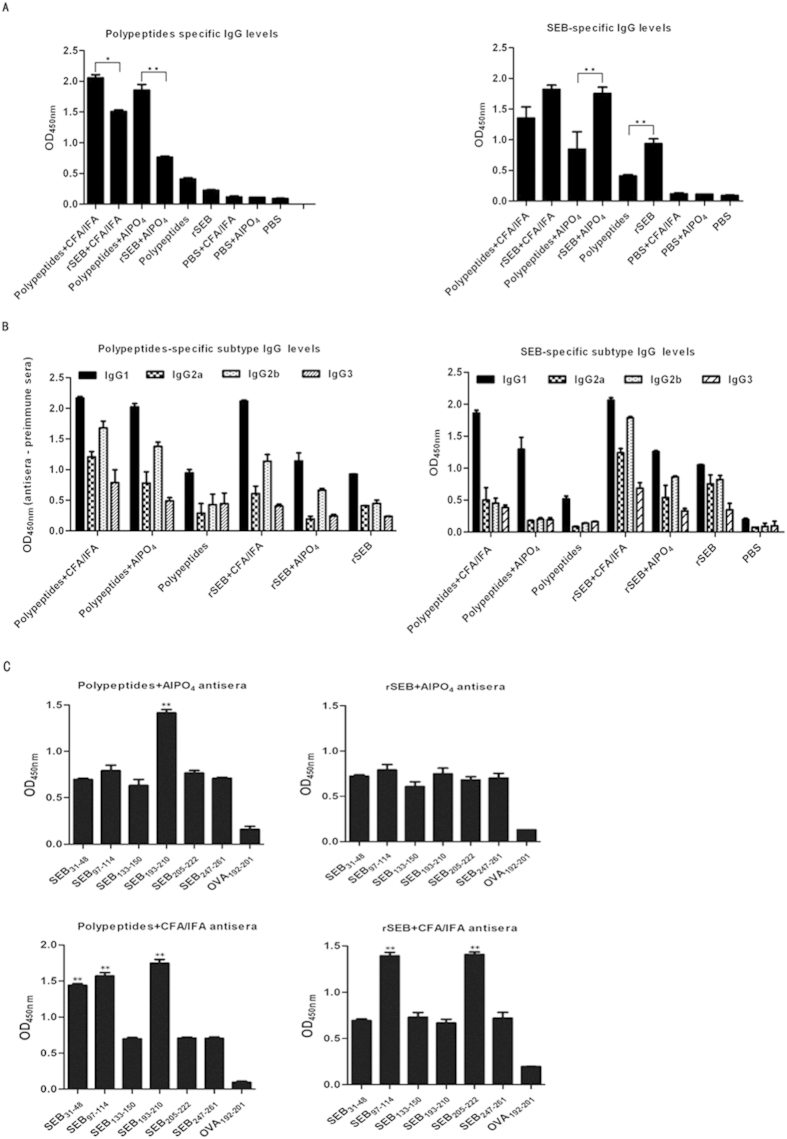
Polypeptides-specific antibody production after MRSA252 challenge. (**A**) Antibody production in Polypeptides or rSEB-vaccinated mice. (**B**) Polypeptides-specific antibody isotype analysis in Polypeptides or rSEB-vaccinated mice. Results are representative of three experiments and data represent the mean ± SD of samples from ten mice. (**C**) The individual immunodominant epitope-specific antibody response following Polypeptides or rSEB vaccination.

To assess the Polypeptides-specific or SEB-specific antibody titre, the sera of immunised mice were examined by ELISA 1 week after the last booster immunisation. Notably, every vaccinated group showed increased levels of specific IgG, while mice immunised with Polypeptides or rSEB plus an adjuvant had significantly higher serum antibody IgG levels. Vaccination of Polypeptides plus CFA/IFA or AlPO_4_ adjuvant also induced a significantly larger SEB-specific antibody response compared to the PBS control groups (P < 0.01) (Fig. 5A).

Serum IgG isotype analysis in Polypeptides- or rSEB-vaccinated mice revealed a mixed IgG1/IgG2b isotype response that was enhanced with the addition of CFA/IFA or AlPO4 adjuvant (Fig. 5B). Moreover, the isotype ratio (IgG1/IgG2b) was found to be >1.0, with low levels of IgG1 and IgG2b found in control sera. IgG3 levels were negligible in all samples analysed. The pattern was found to be similar with all of the adjuvanted vaccine formulations. Given that the protective effect was observed 21 days after infection, the IgG1/IgG2b-biased response may correlate with the high survivals of vaccine-immunized mice against MRSA infection. Additionally, the higher IgG1 levels were found in immunised mice likely indicates that the SEB-specific IgG1 predominates the response against MRSA challenge.

To determine whether antibodies are generated in response to each of the six epitopes present in the Polypeptides vaccine, serum immunodominant peptides specific IgG titres of immunised mice were analysed by ELISA. The response spectrum for the individual epitopes varied across the different vaccination groups. Interestingly, SEB_31–48_, SEB_97–114_, and SEB_193–210_ induced stronger responses in Polypeptides plus CFA/IFA-immunised mice (P < 0.01), whereas SEB_193–210_ induced a stronger response in the Polypeptides plus AlPO4-immunised counterparts (P < 0.01). In comparison, all of the individual epitopes induced a similar level of response in mice immunised with rSEB plus AlPO4 adjuvant (P < 0.01), whereas SEB_97–114_ and SEB_205–222_ induced stronger responses in rSEB plus CFA/IFA-immunised counterparts (P < 0.01; Fig. 5C). Given that the different protective effects were observed, SEB_193–210_ might be a more immunodominant epitope for MRSA infection, while immunisation with the Polypeptides epitope-cocktail may induce a synergistic response against MRSA challenge.

### Polypeptides antisera stimulated the synergetic opsonophagocytosis of MRSA bacteria

**Figure 6:**
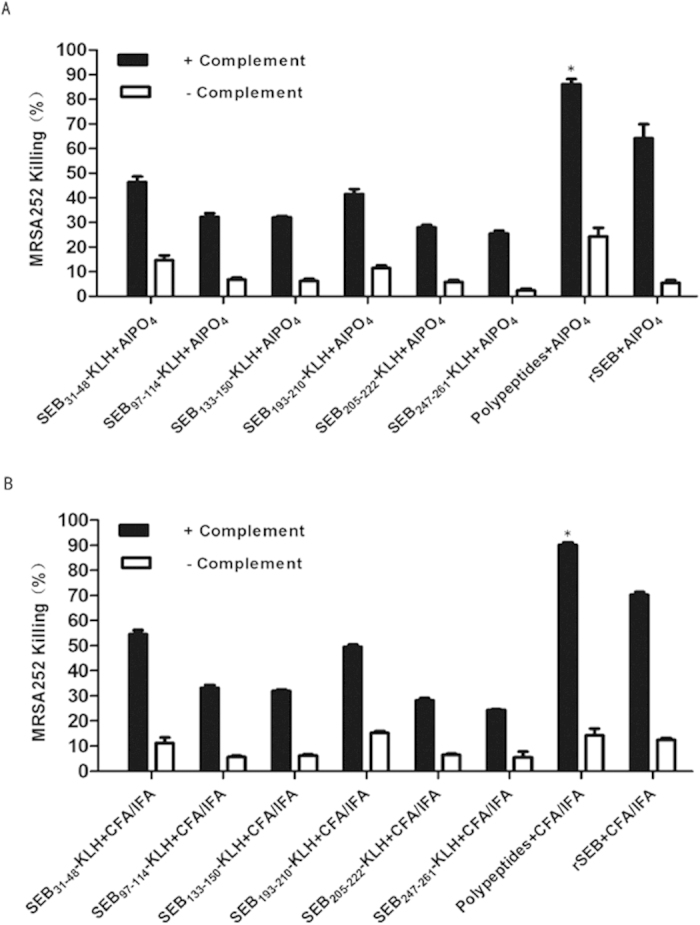
Opsonophagocytosis stimulated by antisera against SEB_31–48_, SEB_97–114_, SEB_133–150_, SEB_193–210_, SEB_205–222_, SEB_247–261_, and Polypeptides. Opsonsonophagocytic killing of MRSA252 in the presence of complement was significantly higher for Polypeptides antisera than for individual immunodominant epitope protein antisera (P < 0.05). Percent killing was defined as the reduction in CFU/mL after 2 h compared with that at time zero. Error bars represent the SEM.

We next wished to determine whether sera from peptide-immunised mice could mediate the opsonophagocytic killing of MRSA bacteria. For this, antibodies against SEB_31–48_, SEB _97–114_, SEB_133–150_, SEB_193–210_, SEB_205–222_, SEB_247–261_, and Polypeptides were raised in mice and analysed for their ability to induce the opsonophagocytic killing of MRSA252. We utilised microtitre-based opsonophagocytic killing assays in the presence of human HL-60 differentiated promyelocytic leukaemia cells, which are commonly used as phagocytic effector cells in opsonophagocytic antibody assays (OPA) routinely used in the *S. aureus* vaccine field^30^,^31^ (Fig. 6). As expected, antibodies against all six individual epitopes induced the opsonophagocytic killing of MRSA; however, the antisera from Polypeptides-immunised mice were significantly more effective at killing MRSA than the individual epitope antisera (P < 0.03).

### Polypeptides protected BALB/c mice against infection by various clinical MRSA isolates

**Figure 7:**
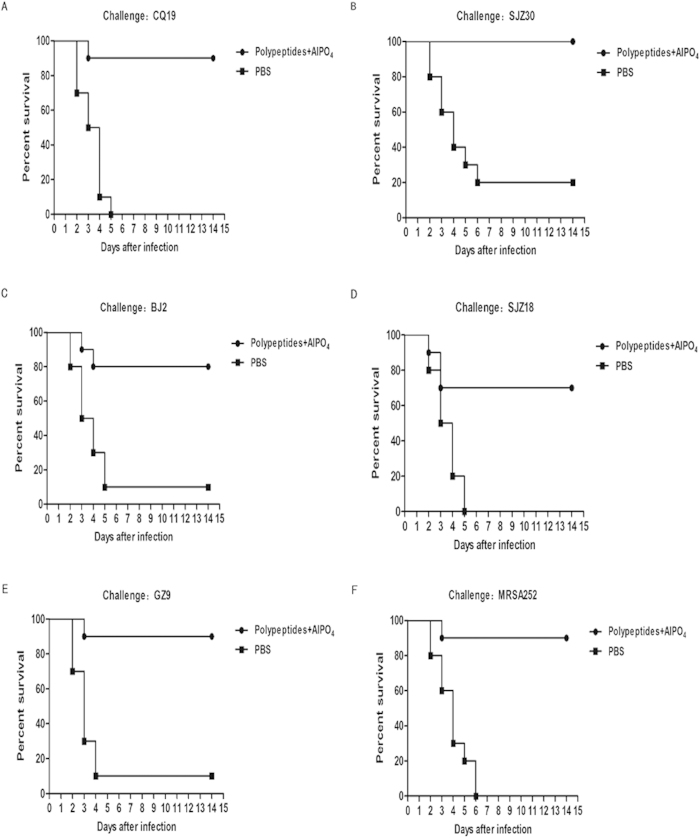
Immunisation with Polypeptides generates protective immunity against lethal challenge with six different clinical MRSA isolates. Survival of Polypeptides-immunised mice following challenge with clinical MRSA isolates (n = 20 for every isolate, of which n = 10 for Polypeptides-immunised mice and n = 10 for PBS-immunised mice). Compared with animals receiving mock-immunization (PBS), the significance of protective immunity generated by the combined vaccine was measured with Fisher’s exact test: (A) CQ19: P <  0.001; (B) SJZ30: P <  0.001; (C) BJ2: P =  0.005; (D) SJZ18: P =  0.003; (E) GZ9: P =  0.001; (F) MRSA252: P <  0.001.

Protection must be achieved against a wide variety of different strains in order for MRSA vaccines to be effective. Thus, we aimed to develop a vaccine effective across a wide range of MRSA isolates by selecting the protective, immunodominant SEB epitopes from MRSA252. Survival analysis revealed that the Polypeptides vaccine afforded significant protection against lethal challenge with all of the five clinical MRSA isolates (CQ19: P < 0.001; SJZ30: P < 0.001; BJ2: P = 0.005; SJZ18: P = 0.003; GZ9: P = 0.001; MRSA252: P < 0.001) (Fig. 7).

## Discussion

MRSA is an increasingly common multi drug-resistant clinical pathogen responsible for skin and soft tissue infections, bacteraemia, endocarditis, and pneumonia^32^. MRSA is highly prevalent in hospitals worldwide, particularly in North and South America, Asia, and Malta, and has become a great threat to public health^33^. Thus, novel vaccines and immunotherapeutic strategies to reduce the incidence and/or mitigate the severity of MRSA infections may yield a significant reduction in morbidity and mortality.

SEB produced by MRSA is considered to be the primary causative agent of staphylococcal toxic shock syndrome^11^. Previous studies have reported that attenuated SEB is fully protective against aerosolised SEB challenge in nonhuman primates^21^ and induced a protective response against *S. aureus* infections in immunised mice, yielding a survival rate of 70%^22^. SEB mutant immunisation (L45R, Y89A, Y94A, and N23K or F44S) with cholera toxin or aluminium hydroxide adjuvants, respectively, protects mice from lethal SEB challenge or *S. aureus* infection^34^. In addition, we previously demonstrated that rSEB confers protection against MRSA252, which produces several enterotoxins^20^. Altogether, these results confirm that SEB as a good vaccine candidate against SEB-producing MRSA infections.

The immunodominant B-cell epitopes are now recognised as key mediators for the induction of humoral immune responses against target antigens. Thus, we suggested that immunisation with whole SEB antigen has hindered the development of effective MRSA vaccines. To design an SEB B-cell immunodominant-epitope-based MRSA vaccine, all protective B-cell immunodominant SEB epitopes should be known. Thus, we used overlapping-peptide ELISAs in combination with computational methods to identify the linear B-cell epitopes within SEB according to the previous study in melanoma^35^. A previous study in our lab mapped three novel immunodominant SEB peptides using rSEB antisera^20^. In the current study, we identified seven dominant response peptides using sera isolated from MRSA-infected mice, including the signal peptide SEB_1–18_ and three novel immunodominant peptides SEB_31–48_, SEB_133–150_, and SEB_193–210_. We also found that the immunodominance of the later three peptides was markedly lower in rSEB immunised mice naïve to MRSA. A recent HIV vaccine study reported that significant differences between the immunodominant epitope patterns are generated in response to immunisation or natural infection^36^. Accordingly, we demonstrated that rSEB immunisation induced immunodominant B-cell epitope responses that differed from those induced by natural MRSA infection.

A complete understanding of protective B-cell epitopes is essential when developing epitope-based vaccines^37^,^38^. Vaccination with long synthetic peptides was previously thought to result in robust immune responses, as demonstrated in several mouse models and clinical studies^39^,^40^. Of the seven immunodominant B-cell epitopes identified in our study, SEB_1–18_ is a signal peptide that is usually cleaved from the mature peptide. We suggested that under certain circumstances—such as being attacked by immune cells—some bacterial cell lyse and release enterotoxins that act as antigens to induce humoral responses (Fig. 1). Nevertheless, since vaccine design usually focuses on antigenic protein coding regions, we focused our study on the other six immunodominant epitopes. SEB-specific IgG is known confer passive protection against diverse *S. aureus* infections^18^. Our study showed that immunisation with the individual immunodominant epitopes induced high levels of epitope-specific IgG and partially protected against MRSA infection (Fig. 2B). Moreover, serum IgG isotype analysis in individual epitope-vaccinated mice showed a mixed IgG1/IgG2b isotype response against MRSA challenge (Fig. 2C), with an isotype ratio (IgG1/IgG2b) >1.0, indicative of a Th2-directed isotype response^41^.

Previous attempts to develop a MRSA vaccine based on a single or multiple antigens have been tested in clinical trials, but none elicited a protective response in humans^8^. A previous review reported that B-cell epitope-based vaccines, which are mainly composed of the protective epitopes, are more effective than those using whole antigens against staphylococcal infections^23^; therefore, this strategy has been extended to vaccines for several other pathogens, such as HIV^27^, respiratory syncytial virus^28^, and *Helicobacter pylori*^29^. While a multiple B-cell epitope-based vaccine has yet to be approved for use in humans, we believe that this strategy may be useful in optimising MRSA vaccine design since every immunodominant epitope in Polypeptides could individually induce a protective antibody response against MRSA. To overcome the limitations of low immunogenicity and protective effects induced by a single-epitope vaccine, we constructed the multiple SEB epitope vaccine, Polypeptides. By analysing survival and bacterial burden in immunised mice, we demonstrated that Polypeptides was more efficacious than the whole rSEB antigen in preventing MRSA infection. The results were also in concordance with the previous study on clumping factor A (ClfA), another *S. aureus* antigen, in which a B-cell epitope of ClfA was effective against *S. aureus*-induced mastitis^42^. In the present study, clinical serological analysis of 31 sera samples from MRSA**-infected patients revealed cross-reactivity with the known peptides SEB_97–114_, SEB_247–261_, and SEB_205–222_ included in the Polypeptides (data not shown). Significantly, this finding indicates that Polypeptides immunisation will likely elicit a protective response in humans. However, it must be noted that polypeptide epitopes that comprise Polypeptides incorporate GST-tags; thus, it is possible that this tag may have additional impact on immune recognition that may contribute to the observed efficacy.

Isotype switching increases antibody efficacy against staphylococcal enterotoxin B-induced lethal shock and *S. aureus* sepsis in mice^43^. The SEB-specific IgG1 monoclonal antibody 20B1 successfully treats sepsis and deep-seated tissue infection caused by SEB-secreting *S. aureus* strains^19^. In addition, IgG1 antibodies can also sensitise *S. aureus* for subsequent killing by antibody- or complement-dependent cytotoxicity, whereas the IgG2 antibody preferentially opsonises bacteria for lysis by FcγRII bearing leukocytes^44^.

Three mechanisms might explain the highly protective capacity of Polypeptides observed in the present study. First, each individual epitope induced a robust, protective antibody response (Fig. 2B). Secondly, antibody class switching to noncomplement-activating IgG1 in response to *S. aureus* is a characteristic Th2 response^45^. CFA/IFA is not approved in humans, while alum, which can be use in humans, is demonstrated a Th2-type adjuvant^46^. We demonstrated that Polypeptides induced a strong, specific IgG1/IgG2b protective response against MRSA infection following immunisations with the Th2-type adjuvant AlPO_4_. Moreover, the isotype ratio (IgG1/IgG2b) was always >1.0, indicative of a Th2-directed isotype response^41^. In future studies, we plan to use an adjuvant approved for human-use, and combine the B-cell vaccine approach with Th2 T-cell-specific epitopes to develop a more efficient MRSA vaccine. Thirdly, many vaccines have also failed because of their inability to induce efficient humoral responses and the propensity of MRSA to evade opsonisation. However, antisera from Polypeptides-immunised mice induced the synergetic opsonophagocytotic killing of MRSA bacteria.

Protection against diverse clinical pathogenic isolates from diverse forms of disease is of crucial importance for broad-spectrum vaccine efforts. Thus, we aimed at developing a vaccine that would be effective against a wide range of SEB-secreting MRSA isolates. Six MRSA strains from distinct clades were selected based on phylogenic SEB gene sequences: BJ2, JN45, STZ30, GZ9, CQ19, and MRSA252 (Supplementary Figure S3). Each MRSA strain was distinct in its geographical origin or clinical symptoms (Supplementary Figure S4). N315 is a highly prevalent Japanese MRSA strain^13^; ST228, also called the South German clone or Italian clone, is prevalent in several central European countries^14^; Mu3 is a vancomycin-resistant MRSA prevalent in Japan^15^; Mu50 is a hospital-acquired MRSA strains prevalent in Japan^16^; and JH1 and JH9 are prevalent MRSA isolates recovered from a single patient during extensive vancomycin therapy^17^. N315, Mu50, JH1, JH9, Mu3, MRSA252, and ST228 are in the same phylogenic clade based on SEB-type; thus, we theorised that an SEB epitope vaccine sufficient to induce protection against MRSA252 might also be effective against these global MRSA strains. In present study, Polypeptides vaccine afforded significant protection against lethal challenge with all of the five clinical MRSA isolates in different clades based on SEB-type; therefore, we suggest that Polypeptides conveys a broad protective effective response against diverse SEB-producing MRSA strains. Moreover, the sequence homology between SEB and SEC varies from 42% to 67%^34^; therefore, our vaccine might be cross-reactive to non-SEB-secreting strains. Previous studies showed that S. aureus protein A (SpA) as an important vaccine candidiant provided mice with elevated protection against MRSA stains USA300 or Mu50 challenge^47^. In present study, we sequenced several genes including SEA, SEB, SEC, SED, SEE, and SpA for the clinical MRSA isolates (Supplementary Figure S4). The spa-type of BJ2 is similar to the known MRSA strain ST5, the spa-type of the CQ19 is similar to the known MRSA strain NJ^48^(data not shown). For MRSA that do not produce SpA, such as SJZ18 and GZ9 (Supplementary Figure S4), Polypeptides vaccine also afforded robust protection.

Although in present study, the epitope vaccine Polypeptides could play a robust protective role against diverse SEB-producing MRSA isolates from diverse diseases, for some of the MRSA strains that do not produce SEB or homological SEC, the protection role could not cover these isolates. Previous studies had showed that a vaccine that stimulated Th1/Th17 response could protect mice against *S. aureus* infections^49^, and antibodies and TH17 cells are the key to an *S. aureus* vaccine^50^,^51^. As in our study, we did not study the cell response of the multiple B-cell epitope-vaccine. Now, we have investigated into screening of the protective T cell epitope in MRSA antigens.

In conclusion, we identified all the immunodominant linear B-cell SEB epitopes in MRSA252, including three novel epitopes. Then, we constructed the recombinant multiple B-cell epitope-vaccine Polypeptides that contains all six immunodominant epitopes and demonstrated its capacity to induce robust IgG1/IgG2b specific response especially SEB-specific IgG1 response to challenge with various clinical MRSA isolates. In addition, all of the immunodominant epitopes in Polypeptides were sufficient to stimulate the synergetic opsonophagocytosis of MRSA via a Th2-directed IgG1 protective response. Since Th1/Th17 response were also reported to play a protective role in *S. aureus* infection, there is likely a nexus of T-cell response and specific antibody isotype-dependent passive immunisation that must be optimised to achieve a maximum level of protective activity.

## Material and Methods

### Ethics Statement

All methods were carried out in accordance with the approved guidelines by the Animal Ethical and Experimental Committee of the Third Military Medical University (Chongqing; permit number 2011–04). Surgeries were performed under sodium pentobarbital anaesthesia, and all efforts were made to minimise suffering. All experimental protocols were approved by the Animal Ethical and Experimental Committee of the Third Military Medical University (Chongqing; permit number 2011–04). All experiments involving the use of human tissue samples were approved by the Human Ethical and Experimental Committee of the Third Military Medical University (Chongqing; permit number 2011–04). The methods were carried out in accordance with the approved guidelines. We had also obtained informed consents from all the subjects.

### Animals, antigens and Bacterial strains

Six- to eight-week-old SPF female BALB/c mice were purchased from the Experimental Animal Center of Third Military Medical University (Chongqing,China). Recombinant mutant SEB (rSEB) was expressed in E. coli and purified as a C-terminal six-histidine-tagged (6 × His) fusion protein, which was previously established^20^. For every immunodominant peptide of SEB, peptide keyhole limpet hemocyanin (KLH) conjugations were performed by the company(ChinaPeptides Co., Ltd). Native SEB was purchased from the Academy of Military Science of China. The recombinant multiple B-cell epitope-vaccine Polypeptides, was designed by arranging the six epitopes, which was expressed in E. coli and purified as a GST-tagged fusion protein (Supplementary Figure S2).

MRSA strains MRSA252 was obtained from the American Type Culture Collection (Manassas, VA, USA). Besides, 13 clinical MRSA strains were used in these studies.Each MRSA strain was distinct in its geographical origin or clinical symptoms (Supplementary Figure S4). The virulence of all of the used MRSA strains in the murine sepsis model verified by pilot studies.

### *Staphylococcus* enterotoxin B epitope mapping

Forty-two synthetic overlapping peptides, which spanned the entire length of the SEB were constructed according to the reported sequence of SEB (NCBI Sequence ID: WP_000764684.1) of MRSA252.These peptides were separately synthesized, beginning with peptide no. 1 at the N-terminus and ending with peptide no. 42 at the C-terminus of SEB (ChinaPeptides Co., Ltd). The peptides consisted of 18 amino acid residues, with an overlap of twelve amino acids each. The negative control peptide, OVA_192–201_ (EDTQAMPFRV), was also synthesized by the same company. The purity of all of the above peptides was expected to be 90% or higher.

The peptides were dissolved in dimethyl sulfoxide (DMSO) at 0.5 mg/mL and diluted in hydrogen bicarbonate buffer (pH 9.6) to 5 μM. Serum samples collected from naïve or rSEB-immunised MRSA-infected BALB/c mice were diluted 1:300. Non-specific binding was prevented by blocking the coated microtiter plates with phosphate buffered saline (PBS, pH 7.4), which contained 5% skim milk, for 1 h. As secondary antibodies, peroxidase-conjugated goat anti-mouseIgG antibodies (Dianova, Hamburg, Germany) were used at a dilution of 1:3000. Optimal peptide concentrations and serum dilutions were determined using serial dilutions. The results of the ELISA were given as absorbance values. The normal values for each peptide were calculated by testing sera from normal mice. The values that were above the mean absorbance value of these sera plus three times the standard deviation were defined as positive.

### Individual immunodominant SEB epitopes or Polypeptides immunisation and MRSA challenge

BALB/c mice were immunised with 100 μg of the six individual immunodominant peptides KLH conjugations or 100 μg Polypeptides or 100 μg rSEB plus AlPO_4_ or CFA/IFA adjuvant, Polypeptides or rSEB alone, AlPO_4_ or CFA/IFA adjuvant alone, or PBS alone at three-week intervals prior to lethal MRSA252 challenge. Two weeks after the last immunisation, mice were infected with MRSA252 (10^9^ CFU) by intravenous tail-vein injection. Immune-serum was collected seven days after the final boost injection and was stored at −20°C until use. Survival was monitored at 8, 16, and 24 h and everyday thereafter for 21 days, at which point the animals were euthanised and the kidneys, livers, lungs, and hearts were harvested to determine bacterial load. The bacterial numbers in the organs were enumerated by preparing organ homogenates in PBS and plating 10-fold serial dilutions on tryptic soy agar (BD Diagnosis System). The colonies were counted after 24 h of incubation at 37 °C. Meantime, the colonization by MRSA was quantified by real-time PCR using the TaqMan method, amplifying mecA of MRSA as previously described^52^. For histopathology, the organs were fixed with 10% phosphate buffered formalin and embedded in paraffin. Four-micrometer thick sections were prepared and stained with hematoxylin and eosin for microscopic examination.

### Immunoglobulin subtyping

The profiles of specific IgG subclasses in antisera were determined by ELISA. Briefly, microtitre plates were coated with Polypeptides or the individual immunodominant epitope 1 μg/well overnight at 4 °C. After blocking with 2% BSA (v/v), Polypeptides or the individual immunodominant epitope-specific antisera were added at a dilution of 1:500 (data at the factor of 1:250, 1:1000 and 1:2000 not shown) and incubated for 1 h at 37 °C. Normal mouse sera (pre-immune sera) served as a negative control. After washing, IgG isotype-specific primary antibodies (goat anti-mouse IgG1, IgG2a, IgG2b and IgG3, purchased from AbD Serotec) were added to the wells at 1:3,000 dilution and incubated for 1 h at 37 °C. After extensive washing, tetramethyl benzidine (TMB) substrate was added for 10 min at room temperature, and the reaction was stopped by addition of 100 μl 2 M sulfuric acid. Endpoint absorbances were read at 450 nm using a microplate reader (Bio-Rad).

### Tissue histology

On day 21 post infection, the livers, spleens, lungs, hearts and kidneys were harvested for the determination of the bacterial burden. The bacterial numbers in the organs were enumerated by preparing organ homogenates in PBS and plating 10-fold serial dilutions on tryptic soy agar (BD Diagnosis System). The colonies were counted after 24 h of incubation at 37 °C. Meantime, the colonization by MRSA was quantified by real-time PCR using the TaqMan method, amplifying mecA of MRSA as previously described^52^. For histopathology, the organs were fixed with 10% phosphate buffered formalin and embedded in paraffin. Four-micrometer thick sections were prepared and stained with hematoxylin and eosin for microscopic examination.

### Antisera-mediated opsonophagocytic killing of MRSA bacteria

Mice were immunised with six individual immunodominant epitope-protein vaccine candidates and the resulting antisera tested for functional activity by *in vitro* opsonophagocytic killing assay (OPK). The microtitre-based OPK assay was described previously^31^. Briefly, differentiated HL-60 cells were incubated with MRSA in the presence of complement and antibody sera (1:2 final dilution) from naïve or immunised mice (n = 7) for 2 h at 37 °C. The percentage of viable MRSA bacteria remaining relative to the initial input was determined by lysis of HL-60 cells and enumeration of bacterial colonies on agar. Control samples included MRSA252 incubated with complement and HL-60 cells (no antisera), MRSA incubated with mouse antiserum or IgG control and complement (no HL-60 cells), or MRSA252 and HL-60 cells only (no complement). The serum used as our complement source did not exhibit any functional effects of anti-teichoic acid antibody contamination. In further support of this, our control mixture consisting of HL-60 cells, MRSA252, and guinea pig serum—but no antibody—exhibited no OPK activity.

### Phylogenetic analysis

Sequence alignments and phylogenetic trees were performed using the MEGA program (version 6.0)^53^. Phylogenies were inferred using the neighbor-joining method and a maximum composite likelihood nucleotide model^54^. The reliability of phylogenetic inference at each branch node was estimated by the bootstrap method with replicates, using the MEGA program. Genotypes were determined according to WHO recommendations. Distances between and within clusters were calculated using the MEGA program.

### Challenge with clinical MSRA isolates

The strains for challenge were selected based on evolutionary analyses using the Maximum Composite Likelihood model conducted in MEGA6^53^,^54^. According to the phylogeny of the human clinical MRSA isolates and SEB sequence-known MRSA strains, five clinical MRSA isolates (not including MRSA252) were selected (Supplementary Figure S3). MRSA252 is a hospital-acquired epidemic human MRSA strain associated with MRSA septicaemia^55^. The human clinical isolates CQ19, SJZ18, BJ2, SJZ30, and GZ9 are sequenced MRSA strains from different regions of China and were isolated as follows: BJ2 was from the sputum of a patient with pneumonia; CQ19 was from the blood of a patient with septicaemia; GZ9 was from the secretion of a patient with traumatic brain injury; and SJZ18 was from the peritoneum dialysate of a patient with renal failure. SJZ30 was from the secretion of a second-degree burn patient with skin infections. Besides MRSA252, all of the five strains were from distinct clades based on SEB gene sequences. Information regarding these clinical MRSA isolates is provided in Supplementary Figure S4. For challenge experiments, mice were immunised with Polypeptides or PBS control, and then challenged by tail-vein injection of solutions containing one of the MRSA strains. Survival analysis was then monitored as described above.

### Statistical analysis

Statistical analyses were performed using GraphPad Prism 5.0 (GraphPad Software). All data were represented as the mean ± standard deviation (S.D.). Data were analyzed using Student’s t-test. *P < 0.05 was considered significant. **P < 0.01 was considered statistically significant. Fisher’s exact test was used to analyze the statistical significance of the lethal challenge data. One-tailed Student’s t tests were performed to analyze the statistical significance of renal abscess data^56^,^57^.

## Additional Information

**How to cite this article**: Zhao, Z. *et al*. Multiple B-cell epitope vaccine induces a *Staphylococcus* enterotoxin B-specific IgG1 protective response against MRSA infection. *Sci. Rep*. **5**, 12371; doi: 10.1038/srep12371 (2015).

## Supplementary Material

Supplementary Information
