# Supplementary material for: Multiple B-cell epitope vaccine induces a Staphylococcus enterotoxin B-specific IgG1 protective response against MRSA infection
Source: Sci Rep. 2015 Jul 23;5:12371. doi: 10.1038/srep12371 (PMC4511869; doi:10.1038/srep12371)
Supplement: Supplementary Information [file srep12371-s1.pdf]

Supplementary Information:

1. **Title of the manuscript:**

Multiple B-cell epitope-vaccine induces SEB specific IgG1 protective response against MRSA infection

2. **Author list:**

Zhuo Zhao [a], He-Qiang Sun [a], Shan-Shan Wei [b], Bin Li [a], Qiang Feng [a], Jiang Zhu [c], Hao Zeng [a], Quan-Ming Zou [a]*, Chao Wu [a]*

[a] National Engineering Research Center of Immunological Products, Department of Microbiology and Biochemical Pharmacy, College of Pharmacy, Third Military Medical University, Chongqing 400038, PR China

[b] Department of Gastroenterology, Xinqiao Hospital, Third Military Medical University, Chongqing 400038, PR China

[c] Department of Pathology, Southwest Hospital, Third Military Medical University, Chongqing 400038, PR China

3. Supplementary figure legends:

Supplementary Figure S1.

The amino acid sequence of the individual immunodominant-epitope in Polypeptides.

Supplementary Figure S2.

Preparation of the multiple epitope vaccine of SEB

A multiple-epitope vaccine of SEB, termed Polypeptides, was designed by arranging the six epitopes in the order of amino acids (31-48)-(97-114)-(133-150)-(193-210)-(205-222)-(247-261) (Supplementary Figure S1). Based on SDS-PAGE, the purified Polypeptides has 366 of amino acids with the molecular weight of 41536.2KDa (Supplementary Figure S2A). Western blot analysis showed that Polypeptides shows good immunogenicity, and in addition, it did not react with normal mouse serum (Supplementary Figure S2B).

(A)Purified Polypeptides expression analysis by 12% SDS-PAGE. Polypeptides was purified by ion exchange chromatography. Lane1, protein Marker; Lane 2, purified Polypeptides.

(B)Western blot. Western blot analysis using primary antibody anti-Polypeptides,

anti-GST sera, normal sera. BALB/c mouse was immunized by Polypeptides in order to produce antiserum. Lane1, antiserum to Polypeptides; Lane 2, anti-GST MAb; Lane 3, normal mouse serum.

Supplementary Figure S3.

Phylogeny of many clinical MRSA isolates and MRSA252 based on SEB gene sequences.

Sequence alignments and phylogenetic trees were performed using the MEGA program (version 6.0)[33]. Phylogenies were inferred using the neighbor-joining method and a maximum composite likelihood nucleotide model[32]. The reliability of phylogenetic inference at each branch node was estimated by the bootstrap method with replicates, using the MEGA program. A maximum likelihood tree based on SEB gene sequences of the 13 clinical MRSA isolates and 8 sequence-known MRSA isolates illustrating the distribution of the SEB toxic activities of each isolate. MRSA strains N315, Mu50, JH1, JH9, Mu3, ECT-R2, MRSA252 and ST228 are sequence-known. The SEB of 13 human clinical MRSA isolates CQ19, KM11, CQ18, CQ7, SJZ40, BJ5, BJ2, JN45, JN64, SJZ30, GZ9, GZ22 and SJZ18 were from different regions of China, and sequenced by the company(TaKaRa, China).The analysis involved 21 nucleotide sequences.The isolates were divided into seven distinct clades based on SEB gene sequences (without significant geographic isolation).

Supplementary Figure S4.

Information of the clinical MRSA isolates

4. Supplementary figures

S1

The individual immunodominant-epitope in Polypeptides	Amino acid sequence
$SEB_{31\text{-}48}$	KASEFTGLMDNMRYLYDD
$SEB_{97\text{-}114}$	NKNIDLFGTNYYYQCYFS
$SEB_{133\text{-}150}$	GGVTEHDGNQIDKNNSTD
$SEB_{193\text{-}210}$	KHKNLYEFNSSPYETGYI
$SEB_{205\text{-}222}$	YETGYIKFIEGNGHSFWY
$SEB_{247\text{-}261}$	VESKSINVEVHLTKK

S2

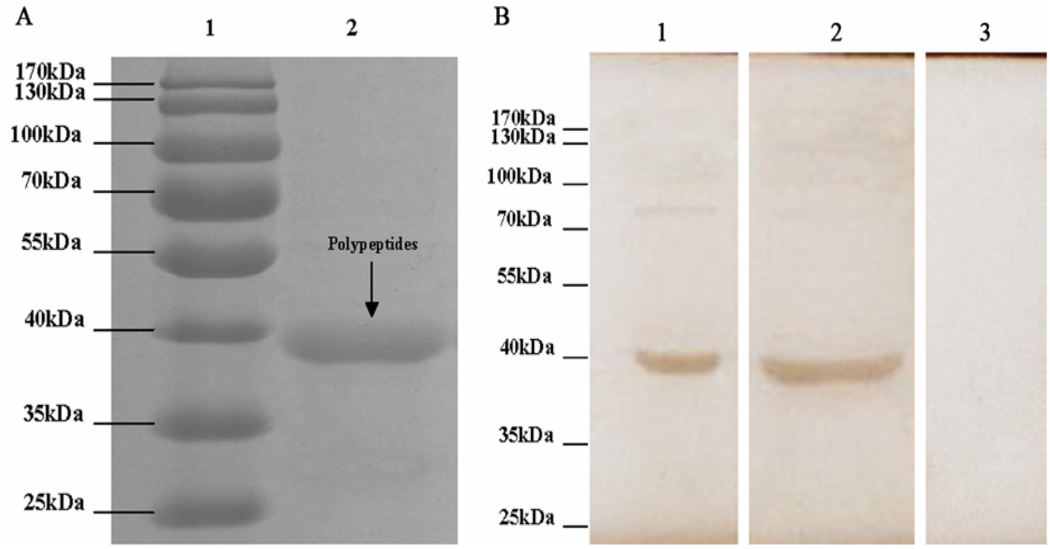

S3

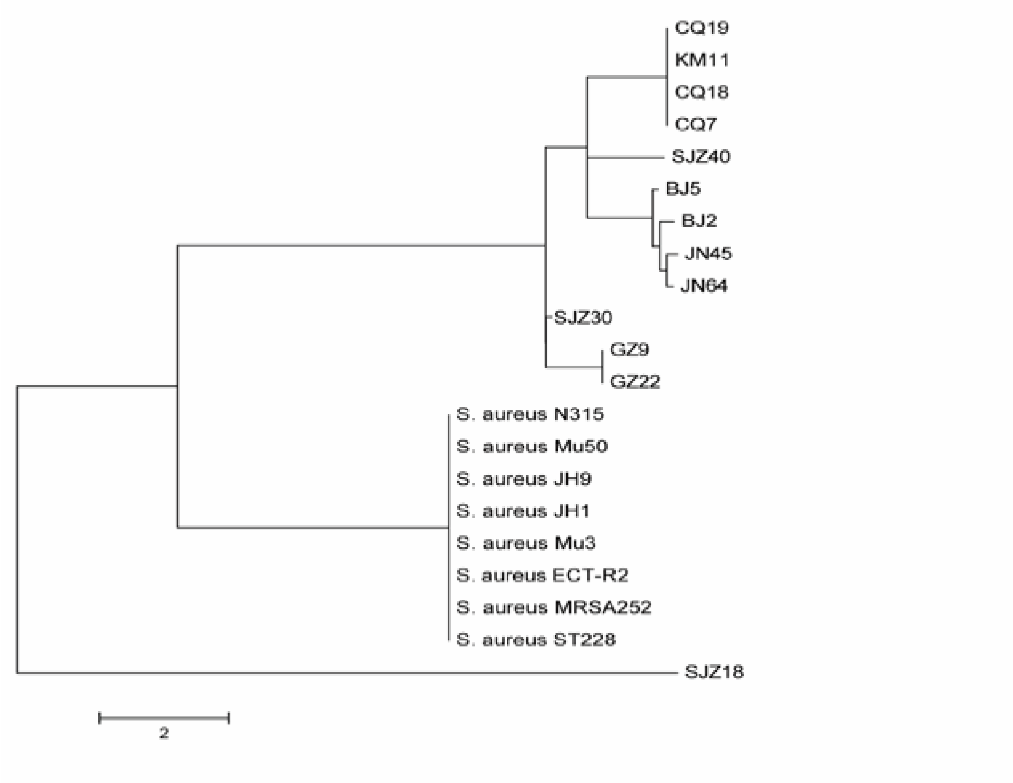

S4

Clinical MRSA isolates	Isolate origin	The type of disease	MecA gene	Enterotoxin gene	Staphylococcus aureus protein A gene
KM11	Secretion	Chest trauma	+	SEB,SEC	+
CQ7	Secretion	Skin infections	+	SEB,SEC	+
CQ18	Blood	Septicaemia	+	SEB,SEE	+
CQ19	Blood	Septicaemia	+	SEB,SEE	+
BJ2	Sputum	Pneumonia	+	SEA,SED,SEE	+
BJ5	Stool	Enteritis	+	SEA,SED,SEE	+
JN45	Peritoneum Dialysate	Renal failure	+	SEB,SED,SEE	+
JN64	Pucture fluid	Mastitis	+	SEB	+
GZ9	Secretion	Traumatic brain injury	+	SEA,SEB,SED,SEE	-
GZ22	Seroperitoneum	Abdominal tumor	+	SEA,SEB,SEC,SEE	+
SJZ18	Peritoneum dialysate	Renal failure	+	SEB,SEC,SEE	-
SJZ30	Secretion	Second-degree burn	+	SEB,SEC	+
SJZ40	Sputum	Pneumonia	+	SEA,SED,SEE	-

"+ "means the gene has been confrimed by PCR analysis."-" means the gene is absent in the isolate.
